# Rheumatism

**Published:** 1870-04

**Authors:** 


### RHEUMATISM.

Persons afflicted with this terrible disease, will thank us for a few hints which, if followed, will tend to alleviate their sufferings or perhaps, result in a perfect cure.

The first thing necessary for the rheumatic patient to do is, to see that he is well enveloped in fine, soft flannel. Not one particle of cotton or linen goods should come in contact with his skin—not even a shirt collar. The sheets upon his bed should be removed, and the patient compelled to lie between fine, woolen blankets. His room should be of an even temperature, day and night. He should sleep alone—at least no one should occupy the same bed with him. His limbs, or the parts most in pain, should be thoroughly rubbed, with a dry hand, morning and evening. No moisture should come in contact with his skin. All sorts of liniments are generally, worse than useless. The principal treatment should be with reference to diet. The patient should avoid everything containing sugar. For this reason vegetables and all kinds of bread should not be eaten. The diet should consist *exclusively* of lean beef, or mutton and eggs. The meat is best broiled (without butter) or roasted. The eggs poached or boiled. Of these articles, he may be allowed to eat freely. Of drink, he may have lemonade without sugar. All the medication necessary is for the patient to apply to his family physician for *potassæ nitr.*, and *opium*.

Our reason for this sort of treatment will appear, when we inform the reader that rheumatism attacks those tissues of the body which are more largely constructed of albumen and fibrin, and that the rheumatic always has an excess of saccharine matter in his blood. Hence the necessity for avoiding such articles of food that supply the blood with sugar, and to partake freely of those that tend to supply albumen and fibrin—the tissues that are undergoing destruction through inflammation.

We have seen many cases of chronic rheumatism recover rapidly, under this treatment after they had applied the usual remedies used in such cases, in vain. We believe it to be the only rational mode of combatting this disease, and therefore, earnestly recommend it to the afflicted.

